# *NOD1* rs2075820 (p.E266K) polymorphism is associated with gastric cancer among individuals infected with *cag*PAI-positive *H. pylori*

**DOI:** 10.1186/s40659-021-00336-4

**Published:** 2021-04-20

**Authors:** Patricio Gonzalez-Hormazabal, Diana Pelaez, Maher Musleh, Marco Bustamante, Juan Stambuk, Raul Pisano, Hector Valladares, Enrique Lanzarini, Hector Chiong, Jose Suazo, Luis A. Quiñones, Nelson M. Varela, V. Gonzalo Castro, Lilian Jara, Zoltan Berger

**Affiliations:** 1 Human Genetics Program, Institute of Biomedical Sciences (ICBM), School of Medicine, University of Chile, 8380453 Santiago, Chile; 2 Department of Surgery, University of Chile Clinical Hospital, 8380456 Santiago, Chile; 3 Department of Surgery, School of Medicine at Eastern Campus, University of Chile, 7500922 Santiago, Chile; 4 Department of Surgery, San Juan de Dios Hospital, 8350488 Santiago, Chile; 5 Department of Surgery, Barros Luco Hospital, 8900085 Santiago, Chile; 6 Institute for Research in Dental Sciences, School of Dentistry, University of Chile, 8380492 Santiago, Chile; 7 Department of Basic-Clinical Oncology, School of Medicine, University of Chile, 8380453 Santiago, Chile; 8 Latin American Network for Implementation and Validation of Clinical Pharmacogenomics Guidelines (RELIVAF-CYTED), Madrid, Spain; 9 Section of Gastroenterology, University of Chile Clinical Hospital, 8380456 Santiago, Chile

**Keywords:** Gastric cancer, Polymorphism, Association study, *H. pylori*, NOD1, E266K

## Abstract

**Background:**

*Helicobacter pylori* is detected by pathogen recognition receptors including toll-like receptors (TLR) and nucleotide-binding oligomerization domain (NOD)-like receptors, eliciting an innate immune response against this bacteria. The aim of this study was to assess if polymorphisms of *TLR2*, *TLR4*, *TLR5*, *NOD1* and *NOD2* genes are associated with gastric cancer, in particular in individuals infected with *H. pylori*.

**Results:**

A case-control study of 297 gastric cancer patients and 300 controls was performed to assess the association of 17 polymorphisms. Analyses performed under the allele model did not find association with gastric cancer. However, *NOD1* rs2075820 (p.E266K) showed association with intestinal-type gastric cancer among *H. pylori* infected subjects (OR = 2.69, 95% CI 1.41–5.13, *p* = 0.0026). The association was not statistically significant in diffuse-type gastric cancer cases (OR = 1.26, 95% CI 0.63–2.52, *p* = 0.51). When the analyses were performed in patients carrying *H. pylori* strains harboring the *cag* pathogenicity island (*cag*PAI), we noticed significant association with *NOD1* rs2075820 (OR = 4.90, 95% CI 1.80–3.36, *p* = 0.0019), in particular for intestinal-type gastric cancer cases (OR = 7.16, 95% CI 2.40–21.33, *p* = 4.1 × 10^− 4^) but not among diffuse-type gastric cancer cases (OR = 3.39, 95% CI 1.13–0.10, *p* = 0.03).

**Conclusions:**

*NOD1* rs2075820 increases the risk of intestinal-type gastric cancer among individuals infected with *H. pylori*, particularly in those harboring the *cag*PAI.

**Supplementary Information:**

The online version contains supplementary material available at 10.1186/s40659-021-00336-4.

## Background

The burden of gastric cancer varies according to the region of the world, with incidence being higher in East Asia (22.4/100,000) followed by Central and Eastern Europe (11.4/100,000 and 9.5/100,000 respectively), and lower in Africa and North America (near to 4.0/100,000) [1]. Risk factors for gastric cancer include, among others, *Helicobacter pylori* infection and genetic factors [2]. *H. pylori* is a bacterium that colonizes the stomach, with a prevalence of 60% in Latin America [3]. This pathogen is associated with peptic ulcers (reported in 1–10% of infected subjects), gastric cancer (0.1–3%) and other extra-gastric diseases [4]. According to the IARC, *H. pylori* is considered as carcinogenic to humans (Group 1). A systematic review and meta-analysis [5] of published studies assessing the association of genetic factors with gastric cancer, described nine polymorphisms with significant association. Many of them were found using a candidate gene approach. Some of these polymorphisms were found to be associated in Asians and others in Caucasians.

The Lauren classification recognizes two histological types of gastric adenocarcinoma: intestinal and diffuse. For the first, Correa has described a precancerous cascade starting with atrophic gastritis, intestinal metaplasia and dysplasia [6]. In fact, atrophic gastritis is related with *H. pylori* and associated with gastric cancer risk [7]. This cascade emphasizes the role of inflammation in the pathogenesis of intestinal-type gastric cancer [8]. In contrast, diffuse-type gastric cancer is poorly differentiated and the underlying biological mechanisms leading to this type of gastric cancer are not fully understood [9]. *H. pylori* is detected by pathogen recognition receptors to elicit an innate immune response against the bacteria [10]. The main pathogen recognition receptors involved in this response include toll-like receptors (TLR) and nucleotide-binding oligomerization domain (NOD)-like receptors.

Ten toll-like receptors have been identified in humans, with TLR2, TLR4 and TLR5 being those involved in the recognition of *H. pylori* (reviewed by [11]). TLR2 and TLR4 recognize bacterial lipopolysaccharide (LPS) resulting in the activation of NF-kB [11]. Flagellin is recognized by TLR5, which induces the activation of a MyD88-dependent pathway [12]. The NOD-like receptor family include intracellular proteins that mediate an innate immune response [13]. NOD1 and NOD2 are NOD-like receptors expressed in epithelial and antigen-presenting cells and recognize *H. pylori* peptidoglycan [14] which enters to the host cell through the type IV secretion system of *H. pylori* [15]. The proteins that compose this bacterial apparatus are encoded by the *cag* pathogenicity island (*cag*PAI), a 40 kb DNA element of the *H. pylori* genome containing approximately 32 genes [16].

Some studies assessing the role of polymorphisms in *TLR2*, *TLR4*, *TLR5*, *NOD1* and *NOD2* in gastric cancer have been published. The associations of *TLR4* rs4986790 (Asp299Gly) (minor allele frequency-MAF = 0.06) and rs4986791 (MAF = 0.04) were found to be statistically significant in meta-analyses [5]. The evidence for association of polymorphisms in *TLR5* [17], *NOD1* and *NOD2* is scarce [18, 19]. Almost all studies were in Eastern countries, mainly China.

The aim of our study was to assess the association of common polymorphisms in *TLR2*, *TLR4*, *TLR5*, *NOD1* and *NOD2* with gastric cancer in *H. pylori* infected subjects. We found that a polymorphism in *NOD1* (rs2075820 p.Glu266Lys) increases the risk of intestinal-type gastric cancer among individuals infected with *H. pylori*, particularly harboring *cag*PAI.

## Results

The genotype count of the analyzed polymorphisms in 297 gastric cancer cases and 300 controls are shown in Additional file 1: Table S1. None of the 17 polymorphisms are associated with gastric cancer under the allele model (Additional file 1: Table S1).

To study whether the association of the polymorphisms depends on the *H. pylori* status of the subject, we performed the analysis on a subgroup of infected patients. One hundred and two cases (39.4%) and 102 controls (49.3%) resulted positive for *H. pylori* infection. In the group of 102 gastric cancer cases and 102 controls we did not observe significant associations (Additional file 2). Nevertheless, after stratification according to histological type, the *NOD1* rs2075820 (p.E266K) polymorphism was associated only among cases with intestinal-type gastric cancer in the unadjusted analysis (OR = 2.69, 95% CI 1.41–5.13, *P* = 2.6 × 10^− 3^). The association was not statistically significant in diffuse-type gastric cancer cases (OR = 1.26, 95% CI 0.63–2.52, *P* = 0.51).

*Helicobacter pylori* peptidoglycan is delivered to the cytoplasm of the epithelial cell through the T4SS injection apparatus encoded by the *cag*PAI, and is recognized by intracellular pathogen recognition receptors such as NOD1 and NOD2. To assess if polymorphisms modify their risk depending on the presence of the *cag*PAI, we carried out the association analysis in 87 gastric cancer patients (85.3%) and 43 controls (42.2%) infected with *cag*PAI-positive strains of *H. pylori*. We noticed significant association with *NOD1* rs2075820 (OR = 4.90, 95% CI 1.80–13.36, *P* = 1.9 × 10^− 3^) (Additional file 2), in particular in intestinal-type gastric cancer cases (OR = 7.16, 95% CI 2.40–21.33, *P* = 4.1 × 10^− 4^) but no among diffuse-type gastric cancer cases (OR = 3.39, 95% CI 1.13–10.10, *P* = 0.03). Table 1 summarizes the results of the stratified analyses of *NOD1* rs2075820. Therefore, the results suggest that this polymorphism confers increased risk for gastric cancer only among patients infected with *H. pylori*, in particular those infected with *cag*PAI-positive strains.

*NOD1* rs2075820 is a non-synonymous polymorphism that replaces glutamic acid, a negatively charged amino acid, with lysine, a positively charged amino acid, at residue 266 of NOD1 protein (p.E266K). Additional file 3: Figure S1 shows a multiple sequence alignment of NOD1 from different species. Position 266 is relatively conserved, and glutamic acid or aspartic acid is present at this residue in almost all species, suggesting that this residue corresponds to a negatively-charged amino acid. SIFT and PolyPhen2 were used to assess if the polymorphism affects the function of the encoded protein. The score was 0.043 for SIFT, which is considered “damaging”, and 0.897 for PolyPhen2 classified as “possibly damaging”. Since the crystal structure of NOD1 is not available, we modeled *ab initio* its structure to infer whether the polymorphism alters the structure of the protein. Residue 266 lies in a alpha helix (Additional file 4: Figure S2). According to the *ab initio* modeled protein (Additional file 4: Figure S2), lysine at residue 266 does not modify the structure of the alpha helix compared to the protein with aspartic acid at 266. Nevertheless, a detailed inspection of the structure reveals a change in the orientation of lateral chains at residues 266 and 267 (Additional file 4: Figure S2). Taken together, it is possible that this polymorphism affects the function of NOD1.

**Table 1:** Association of *NOD1* rs2075820 with gastric cancer

	Cases	Controls	OR (95% CI)^a^	*P*-value^a^	OR (95% CI)^b^	*P*^b^
All subjects						
All samples	297^c^		1.29 (0.96–1.72)	0.092	1.29 (0.92–1.72)	0.095
Intestinal-type	157		1.08 (1.08–2.14)	0.016	1.55 (1.10–2.20)	0.013
Diffuse-type	140		1.07 (0.74–1.55)	0.732	1.29 (0.96–1.72)	0.095
*H. pylori*-positive						
All samples	102	102	1.88 (1.08–3.24)	0.02	1.70 (0.96–3.00)	0.07
Intestinal-type	50	102	**2.69 (1.41–5.13)**	**2.6 × 10**^**−3**^	2.50 (1.26–4.96)	0.01
Diffuse-type	52	102	1.26 (0.63–2.52)	0.51	1.23 (0.61–2.48)	0.56
*H. pylori cag* PAI-positive						
All samples	87	43	**4.90 (1.80–13.36)**	**1.9 × 10**^**−3**^	4.32 (1.57–11.89)	4.6 × 10^−3^
Intestinal-type	42	43	**7.16 (2.40–21.33)**	**4.1 × 10**^**−4**^	**6.77 (2.18–21.02)**	**9.5 × 10**^**−4**^
Diffuse-type	45	43	3.39 (1.13–10.10)	0.03	3.20 (1.06–9.67)	0.04

^a^Unadjusted

^b^Adjusted for sex, principal component (PC)1 and PC2. OR: odds ratio. 95% CI: 95 % confidence interval. Statistically significant results are shown in bold (Significance level after Bonferroni’s correction < 2.9 × 10^−3^)

^c^Two cases with no-defined adenocarcinoma

## Discussion

In the present study we assessed the role of polymorphisms in genes encoding pathogen recognition receptors in gastric cancer risk. We also aimed to analyze whether the risk changes among patients infected with *H. pylori*, in particular those with strains harbouring the *cag*PAI. The *NOD1* rs2075820 polymorphism (p.E266K) was not associated with gastric cancer, nevertheless, the risk conferred by this SNP was evident only among patients carrying *cag*PAI positive *H. pylori* strains.

Little is known about the association of *NOD1* rs2075820 with gastric cancer. Wang et al. [19] did not find association with gastric cancer in Chinese population. However, they found an increased risk to diffuse-type gastric cancer among individuals infected with *H. pylori* in the recessive model (OR = 1.89 [95% CI 1.07–3.32]) but the risk was not statistically significant in the dominant model (OR = 0.60 [95% CI 0.21–1.70]). The association with intestinal-type gastric cancer was not reported. We were not able to replicate a statistically significant result for the recessive model because just one case homozygous for the A allele was observed in diffuse-type gastric cancer cases (n = 52) and again only one case among the controls (n = 102) infected with *H. pylori*. Atrophic gastritis and intestinal metaplasia are premalignant lesions for gastric cancer. Among cagA seropositive patients, Kara et al. [20] found that carriers of allele A of *NOD1* rs2075820 had a significantly increased risk for antral atrophy and metaplasia. In the study by Kim et al. [21], healthy subjects infected with *H. pylori* and homozygous for the risk allele (A) of *NOD1* rs2075820 had a high gastritis score compared with carriers of the G allele. This effect was not observed among non-infected subjects. In addition, the IL-8 mRNA level of the AA genotype was significantly higher than for GA and GG genotypes among subjects infected with *H. pylori* harboring intact *cag*PAI [21]. Those studies suggest that *H. pylori* influence the association of *NOD1* rs2075820 with preneoplasic lesions of intestinal-type gastric cancer.

Some studies have shed light on the role of NOD1 in gastric carcinogenesis. In an in vivo experiment, intestinal metaplasia was more frequently seen in tissue from NOD1-deficient mice (−/−) infected with *H. pylori* as compared to NOD1-intact mice [22]. The proportion of mice with intestinal metaplasia was low in non infected mice. They demonstrated in vitro that expression of *cdx2*, a protein involved in the normal differentiation of the intestinal epithelium, is higher in NOD1-deficient infected mice than in NOD1-intact infected mice. Uninfected mice do not express *cdx2*. *In vitro* analyses suggest that NOD1 is a negative regulator of cdx2 expression [22]. In another *in vivo* study in mice, the preactivation of NOD1 previous to infection by *H. pylori* reduces the frequency of adenocarcinoma and inflammation score [23]. A recently published article [24] shows that the prevalence of gastric dysplasia in INS-GAS NOD1 −/− infected mice was significantly higher than INS-GAS NOD1 +/+ mice. The above studies suggest that a diminished function of NOD1 contributes to the development of intestinal-type gastric cancer in infected subjects. In the case of *NOD1* rs2075820, we hypothesize that it partially reduces the function of the NOD1 protein.

Our *in silico* analysis of the possible consequence of the amino acid exchange at the residue 266 induced by the *NOD1* rs2075820 polymorphism was not fully conclusive. This residue lies in the central nucleotide-binding domain termed “NACHT” [25]. It is hypothesized that a crucial step in NOD1 activation is the oligomerization of this domain that acts as a platform for binding of adaptor molecules and effector proteins, resulting in an inflammatory response [26]. We propose that the change of a negatively-charged amino-acid to a positively-charged one, together with the possible change in the orientation of lateral chains at residues 266 and 277, could affect the interaction of effector proteins and adaptor molecules with the NACHT domain. The consequence of *NOD1* rs2075820 at the functional and structural level requires further investigation.

The present study has some limitations. It does not necessarily represent the general population of Chile since it is a multi-centric hospital-based study. The sample size is small. Data regarding environmental risk factors were not available, which did not allow us to analyze gene–environment interactions. We did not adjust for age due to the low prevalence of gastric cancer in this study [27].

## Conclusions

Our results indicate that *NOD1* rs2075820 increases the risk of intestinal-type gastric cancer among individuals infected with *H. pylori*, particularly for those carrying the *cag*PAI. This could begin to explain the complex interaction between host genetic factors and *H. pylori* infection.

## Methods

### Subjects

The study included 299 cases (195 men and 104 women) diagnosed with gastric adenocarcinoma according to the histopathological report. The median age of diagnosis was 66 years, and ranged from 25 to 93. Patients were recruited at the time of surgical resection between 2001 and 2018 from the following hospitals in Santiago, Chile: University of Chile Clinical Hospital and Biobanco de Tejidos y Fluidos de la Universidad de Chile (BTUCH), Salvador Hospital, Barros Luco Trudeau Hospital, San Juan de Dios Hospital and Military Hospital of Santiago. Tumors were classified as intestinal-type or diffuse-type according to the Lauren’s classification. The control group was composed of 301 individuals (179 men and 122 women, median age 53 years, ranging from 18 to 82) with no personal history of cancer. Blood samples were collected in EDTA vacutainers for all participants. A sample of gastric mucosa was obtained from 259 cases and 207 controls. For cases, the sample was taken from corpus distant to the tumor, immediately from the resected stomach prior to the histopathological procedure. In the case of controls, a fresh sample was obtained from antrum and corpus, in patients who underwent an upper gastrointestinal endoscopy prescribed by a physician. This study was approved by the institutional review board of University of Chile School of Medicine (#045/2015) and was performed in accordance with the Declaration of Helsinki. All participants gave their written informed consent.

### Genotyping

Genomic DNA was isolated from blood samples using the salting out and Proteinase K method, or according to the protocol described by Chomczynski and Sacchi [28] was further purified using Monarch PCR and DNA cleanup columns (New England Biolabs, USA). Single nucleotide polymorphisms (SNPs) were genotyped at the Human Genomics Facility (HuGe-F) in Erasmus MC, Netherlands, by Infinium Global Screening Array-24 BeadChip (Illumina, USA) according to the manufacturer’s protocol. Quality control of the genotyping data was performed following the guidelines in Anderson et al. [29] using PLINK 1.9 [30]. All studied samples and analyzed SNPs passed the quality control test. SNPs of *TLR2*, *TLR4*, *TLR5*, *NOD1* and *NOD2* genes were selected according to the following criteria: (1) positioned from 5 kb upstream from the transcription start site to 5 kb downstream from the stop triplet according to the GRCh37 assembly of human genome; (2) minor allele frequency (MAF) > 0.10 and (3) with no departure from Hardy-Weinberg equilibrium in the studied population (p < 0.01). We excluded rs5030728, rs4722988, rs2111234, rs2076756, rs4785224 and rs5743266 because they are in linkage disequilibrium (LD) (r^2^ > 0.8) with rs2770150, rs62447420, rs8057341, rs17313265, rs11647841 and rs2066842, respectively. Additional file 5: Table S2 describes the list of 17 polymorphisms analyzed in this study.

### Detection of *H. pylori* and *cag* pathogenicity island

Analyses were performed using DNA from gastric mucosa isolated with FavorPrep Tissue DNA Extraction Mini Kit (Favorgen Biotech Corp, Taiwan, China). Samples were assessed for the presence or absence of *H. pylori* DNA by amplification of the 16S rRNA gene by 5′ exonuclease assay as described by Kobayashi et al. [31]. *cagE* gene was used as a marker of the presence of the *cag*PAI, and its detection was performed by 5′exonuclease assay. Primers were: F: 5′TGTGCTTGTAGCTCTTGGATTC-3′ and R: 5′-TCATGAACGCTTTGTTTTTCAC-3′ and the probe corresponds to 5′-[FAM] CTTTATCAAAGAATGGAGCGAGCGATG-3′ [BHQ1]. Primers and probes were synthesized by Macrogen Inc. (Korea). The 5′exonuclease assay was carried out using 5X HOT FIREPol Probe qPCR Mix Plus (ROX) (Solis BioDyne, Estonia) according to the manufacturer’s directions in a StepOne Real Time PCR system (Applied Biosystems, USA). Samples with a negative result in the *cagE* gene assay were subject to further detection of *cag*PAI empty site by PCR using the primers: 5′-ACATTTTGGCTAAATAAACRYTG-3′ and 5′-CACGCATTTTCCCTTRATC-3′, which amplify a fragment of 532 bp for *cag*PAI-negative samples. The reaction mixture was set up using RBC TaqDNA Polymerase (RBC Bioscience, Taiwan, China) and PCR reagents according to the manufacturer’s instructions. Reaction conditions for PCR were as follows: initial denaturation at 94 °C for 3 min, 40 cycles of denaturation at 94 °C for 30 s, annealing at 58 °C for 30 s, elongation at 72 °C for 45 s, and final elongation at 72° for 2 min. The product was analyzed in 1.5% agarose gel electrophoresis.

### Prediction of functional consequences of *NOD1* rs2075820 polymorphism

The multiple sequence alignment for NOD1 from different species was performed using PRALINE (http://www.ibi.vu.nl/programs/pralinewww/). Sequences were retrieved from UniProt with the following accession codes: *Homo Sapiens* (NP_006083.1), *Callithrix jacchus* (F7GYY3), *Heterocephalus glaber* (G5BRM7), *Equus caballus* (F7AF18), *Rattus norvegicus* (D4ADT7), *Mus musculus* (Q8BHB0), *Sus scrofa* (B0FSM7), *Canis lupus familiaris* (E2R9L3), *Bos Taurus* (E1B7V7), *Loxodonta africana* (G3T781), *Ailuropoda menaloleuca* (G1LJ87), *Myotis lucifugus* (G1NWX5), and *Oryctolagus cuniculus* (G1SNK2). SIFT [32] and PolyPhen-2 [33] were used to predict the possible impact of the amino acid substitution. The structure of NOD1 was predicted using the I-TASSER server [34] using the protein sequence with accession number NP_006083, and the resulting .pdb file was submitted to Missense3D [35] to model the structure of the mutant protein.

### Statistical analyses

Statistical analyses were performed using PLINK 1.9 [30]. The exact test was used to detect departures from the Hardy-Weinberg equilibrium. The set of autosomal genotypes, obtained from Infinium Global Screening Array, was pruned from extended regions of high LD (r^2^ > 0.2) using indep-pairwise to obtain a set of 184,909 autosome SNPs. This set was submitted for principal component analysis (PCA) using pca. Principal component (PC) 1 and PC2 were used as estimates of population stratification [36]. The plot of the distribution of PC1 and PC2 among cases and controls is shown in Additional file 6: Fig. S3. A logistic regression analysis was performed to assess association of SNPs under the allele model, unadjusted or adjusting for sex, PC1 and PC2. Fisher’s exact test of independence was used to compare genotype distribution between cases and controls. A *p*-value < 2.9 × 10^− 3^ was considered statistically significant according to Bonferroni’s correction for multiple comparisons.

## Supplementary Information

**Additional file 1. **Genotype count of studied polymorphisms among gastric cancer cases and controls.

**Additional file 2. **Association analysis of the studied SNPs.

**Additional file 3. **Multiple sequence alignment of *NOD1* from different species. 

**Additional file 4. **Analysis of the consequences of p.E266K on NOD1 protein.

**Additional file 5. **Description of the 17 SNPs analyzed in this study.

**Additional file 6. **Distribution of Principal Component 1 and Principal Component 2 among gastric cancer cases and controls.

## Data Availability

The datasets used and/or analyzed during the current study are available from the corresponding author on reasonable request.

## Abbreviations

TLR: Toll-like receptor
NOD: Nucleotide-binding oligomerization domain
OR: Odds ratio
cagPAI: *cag* pathogenicity island
LPS: Lipopolysaccharide
MAF: Minor allele frequency
SNP: Single nucleotide polymorphism
PC1: Principal component 1
PC2: Principal component 2
