# Supplementary material for: NOD1 rs2075820 (p.E266K) polymorphism is associated with gastric cancer among individuals infected with cagPAI-positive H. pylori
Source: Biol Res. 2021 Apr 20;54:13. doi: 10.1186/s40659-021-00336-4 (PMC8056668; doi:10.1186/s40659-021-00336-4)
Supplement: Supplementary file 1 — Additional file 1. Genotype count of studied polymorphisms among gastric cancer cases and controls. [file 40659_2021_336_MOESM1_ESM.pdf]

Table S1. Genotype count of studied polymorphisms among gastric cancer cases and controls.

Gene	rsID	Genotype count[1]				*P*-value [2]	*P*-value [3]	*P*-value [4]
		Gastric Cancer	Intestinal-type	Diffuse-type	Controls			
NOD1	rs2970498	28/126/145	11/65/81	17/61/62	31/134/136	0.71	0.33	0.85
	rs2075820	11/104/184	7/61/89	4/43/93	9/86/206	0.22	0.04	0.92
	rs62447420	14/107/178	5/55/97	9/52/79	19/123/159	0.23	0.12	0.75
	rs2709803	8/93/198	4/54/99	4/39/97	6/79/216	0.34	0.14	0.75
NOD2	rs7194886	28/107/164	14/54/89	13/53/74	38/121/142	0.14	0.14	0.46
	rs8057341	64/143/92	30/75/52	33/67/40	86/146/69	0.04	0.02	0.35
	rs11647841	26/114/159	12/61/84	13/53/74	36/131/134	0.08	0.13	0.26
	rs2066842	7/66/226	3/32/122	4/33/103	4/82/215	0.2418	0.23	0.36
	rs17313265	8/69/222	3/34/120	5/34/101	4/83/214	0.2715	0.32	0.24
	rs3135499	27/117/155	13/61/83	13/56/71	37/137/127	0.05	0.08	0.23
TLR2	rs3804099	32/136/131	15/78/64	17/57/66	41/125/135	0.44	0.20	0.89
	rs7656411	16/99/184	11/47/99	5/51/84	23/110/168	0.28	0.31	0.26
TLR4	rs2770150	23/112/164	10/66/81	13/45/82	20/135/146	0.18	0.81	0.03
	rs1554973	9/77/213	4/41/112	5/36/99	7/79/215	0.90	1.00	0.76
	rs7037117	15/122/162	6/66/85	9/56/75	20/114/167	0.58	0.40	0.93
	rs913930	31/128/140	13/74/70	18/52/70	29/147/125	0.33	0.79	0.07
TLR5	rs75977922	25/130/144	10/70/77	15/59/66	45/133/123	0.02	0.02	0.35

[1] aa/Aa/AA, being "A" the mayor allele and "a" the minor allele. [2] Gastric cancer versus controls, Fisher's exact test of independence. [3] Intestinal-type versus controls, Fisher's exact test of independence. [4] Diffuse-type versus controls, Fisher's exact test of independence.
