# Supplementary material for: NOD1 rs2075820 (p.E266K) polymorphism is associated with gastric cancer among individuals infected with cagPAI-positive H. pylori
Source: Biol Res. 2021 Apr 20;54:13. doi: 10.1186/s40659-021-00336-4 (PMC8056668; doi:10.1186/s40659-021-00336-4)
Supplement: Supplementary file 3 — Additional file 3. Multiple sequence alignment of NOD1 from different species. [file 40659_2021_336_MOESM3_ESM.pdf]

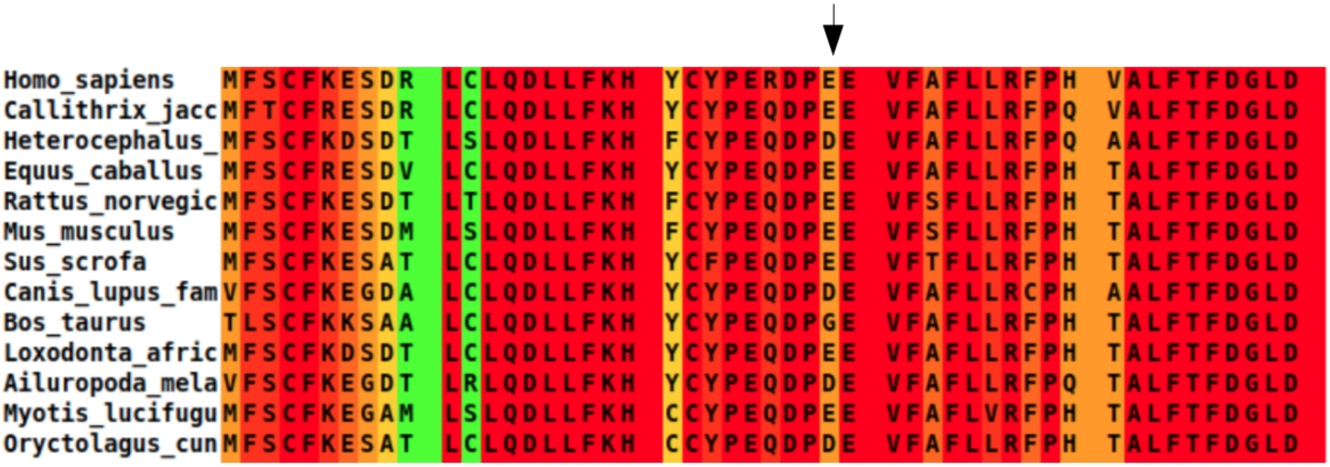

Figure S1. Multiple sequence alignment of *NOD1* from different species. Arrow indicates residue 266.
