# Supplementary material for: NOD1 rs2075820 (p.E266K) polymorphism is associated with gastric cancer among individuals infected with cagPAI-positive H. pylori
Source: Biol Res. 2021 Apr 20;54:13. doi: 10.1186/s40659-021-00336-4 (PMC8056668; doi:10.1186/s40659-021-00336-4)
Supplement: Supplementary file 4 — Additional file 4. Analysis of the consequences of p.E266K on NOD1 protein. [file 40659_2021_336_MOESM4_ESM.pdf]

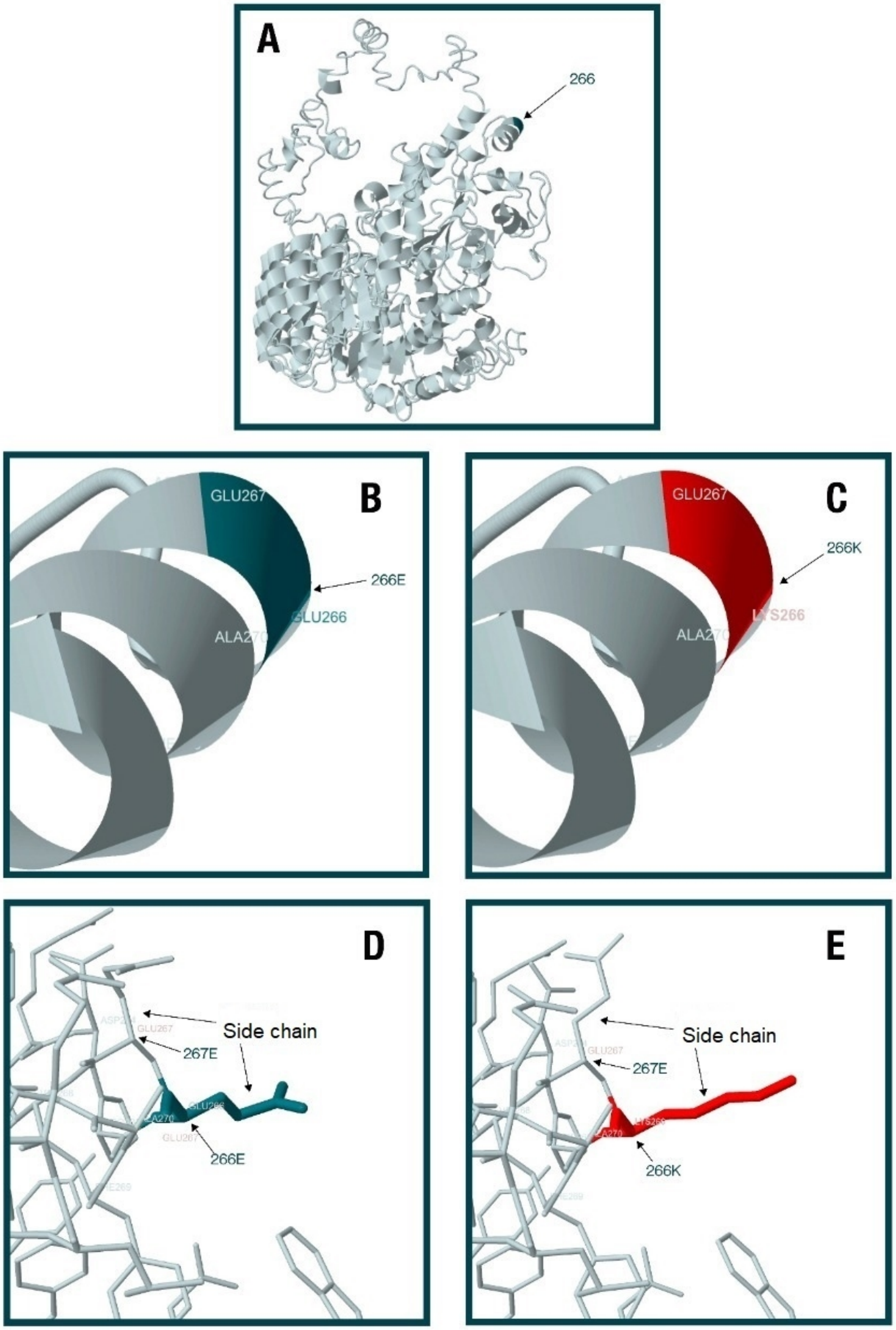


**Figure S2. Analysis of the consequences of p.E266K on NOD1 protein.** A. Modeled structure of NOD1. Lysine at residue 266 (C) does not modify the structure of the corresponding alpha helix compared to glutamic acid -E- at residue 266 (B). The orientation of the lateral chains at residues 266 and 267 with glutamic acid at residue 266 (D) differs compared to the presence of lysine at residue 266 (E).
