# Supplementary material for: NOD1 rs2075820 (p.E266K) polymorphism is associated with gastric cancer among individuals infected with cagPAI-positive H. pylori
Source: Biol Res. 2021 Apr 20;54:13. doi: 10.1186/s40659-021-00336-4 (PMC8056668; doi:10.1186/s40659-021-00336-4)
Supplement: Supplementary file 5 — Additional file 5. Description of the 17 SNPs analyzed in this study. [file 40659_2021_336_MOESM5_ESM.pdf]

Table S2. Description of the 17 SNPs analyzed in this study.

Gene	rsID	HGVS	[1]	Assesed allele	Allele frequency AMR [2]	Allele frequency in controls, present study	HWE *p*-value
NOD1	rs2970498	NM_001354849.1:c.2454-2359G>A	C/T	T	0.30	0.32	1.00
	rs2075820	NM_001354849.1:c.796G>A	G/A	A	0.18	0.17	1.00
	rs62447420	NM_001354849.1:c.-351-967A>G	T/C	C	0.22	0.27	0.66
	rs2709803	NM_001354849.1:c.-352+7688C>T	G/A	A	0.17	0.15	0.82
NOD2	rs7194886	NC_000016.9:g.50725193C>T	C/T	T	0.30	0.33	0.19
	rs8057341	NM_001293557.1:c.460-3786A>G	A/G	G	0.52	0.52	0.64
	rs11647841	NM_001293557.1:c.566-1138G>A	G/A	A	0.30	0.34	0.61
	rs2066842	NM_001293557.1:c.721C>A	C/T	T	0.17	0.14	0.48
	rs17313265	NM_001293557.1:c.2381+1420C>T	C/T	T	0.17	0.14	0.48
	rs3135499	NM_001293557.1:c.*397A>C	A/C	C	0.33	0.35	0.80
TLR2	rs3804099	NM_001318787.1:c.597T>C	T/C	C	0.33	0.34	0.20
	rs7656411	NM_001318787.1:c.*1241T>G	T/G	G	0.24	0.26	0.37
TLR4	rs2770150	NC_000009.11:g.120463139A>G	T/C	C	0.23	0.29	0.26
	rs1554973	NC_000009.11:g.120480812T>C	T/C	C	0.19	0.15	0.82
	rs7037117	NC_000009.11:g.120483663A>G	A/G	G	0.30	0.25	0.76
	rs913930	NC_000009.11:g.120484009G>A	C/T	T	0.27	0.34	0.24
TLR5	rs75977922	NC_000001.10:g.223281096T>C	T/C	C	0.23	0.36	0.45

HSVG: Human Genome Variation Society nomenclature, [1] mayor allele/minor allele defined according to GRCh37 human genome assembly, [2] data from Ad Mixed Americans (AMR) deposited in 1000 Genomes Project Phase 3. HWE: Hardy-Weinberg Equilibrium
