# Supplementary material for: NOD1 rs2075820 (p.E266K) polymorphism is associated with gastric cancer among individuals infected with cagPAI-positive H. pylori
Source: Biol Res. 2021 Apr 20;54:13. doi: 10.1186/s40659-021-00336-4 (PMC8056668; doi:10.1186/s40659-021-00336-4)
Supplement: Supplementary file 6 — Additional file 6. Distribution of Principal Component 1 and Principal Component 2 among gastric cancer cases and controls. [file 40659_2021_336_MOESM6_ESM.pdf]

Figure S3. Distribution of Principal Component 1 and Principal Component 2 among gastric cancer cases and controls.

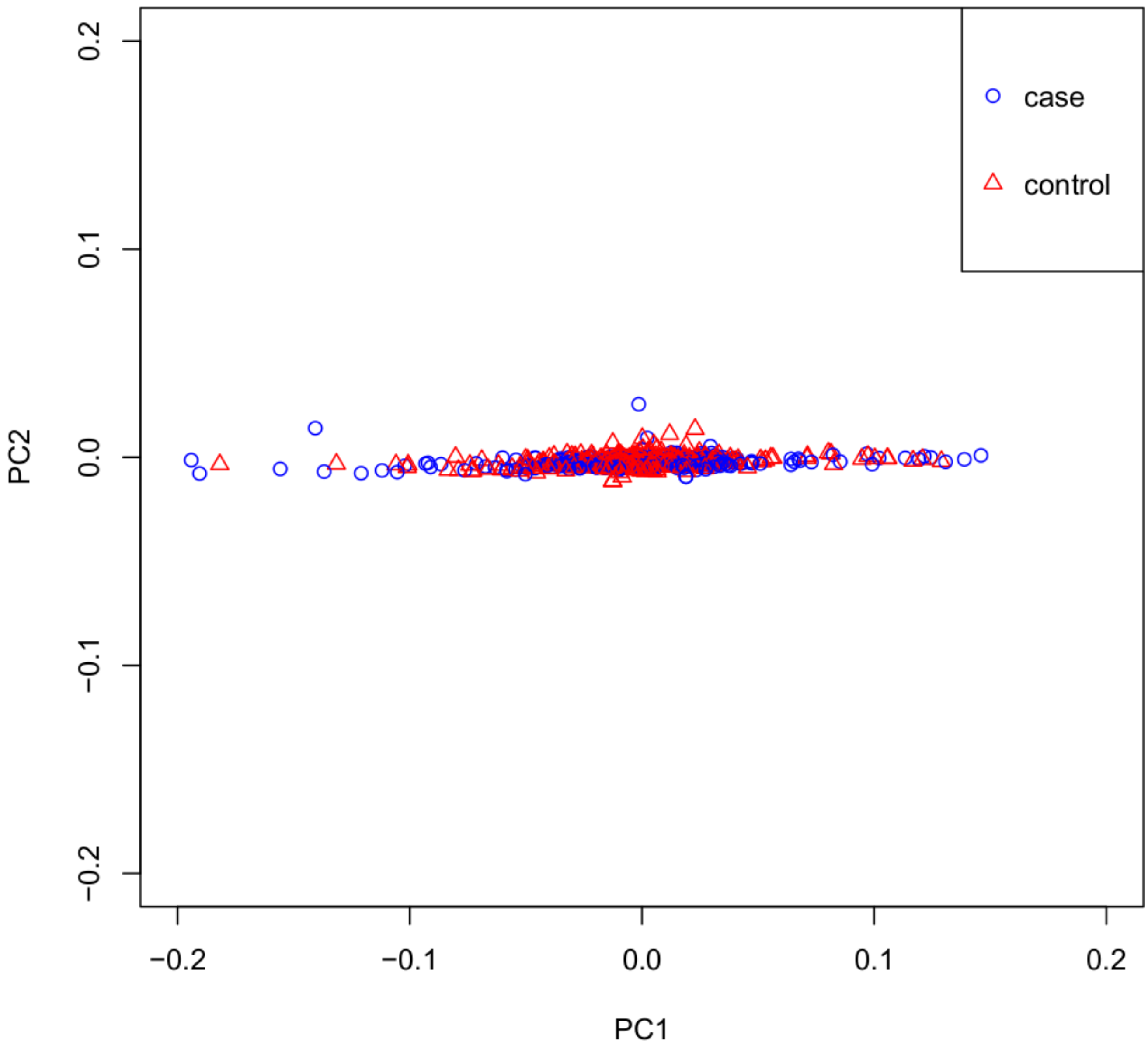

PC: Principal Component.
